# Visiting Committee's Proposals for a Central Body for Discussion

**Published:** 1919-02-01

**Authors:** 


### THE ASYLUMS OF ENGLAND AND WALES.

### Visiting Committee's Proposals for a Central Body for Discussion.

THE Visiting Committees of the asylums in England and Wales are organising themselves into a central body to discuss problems that interest them all in common; and the first meeting of the conference was held on October 29 last. It is adjourned until February 5 next, when the conference will meet at the Guildhall in London. The new body is analogous to the Association of County Councils, and if its deliberations are conducted in the same spirit, it is likely to be equally powerful. Matters on the agenda for the adjourned conference are very important, and include the following:—

1. That the Board of Control be reconstituted so as to include an architect, an engineer, and a layman, each of whom shall have special knowledge and practical experience of mental hospital work so far as it concerns his department.

2. That the medical superintendents of mental hospitals be relieved by legislation, and in practice

### The Asylums of England and Wales—(*continued*).

of as much as possible of their administrative, clerical, and routine duties.

With respect to the first of these resolutions, it is difficult to see that any advantage would accrue to the Board of Control by having an architect and an engineer included among its members, instead of having, as at present, the power of consulting an architect and an engineer who have other work to do. The Board of Control has, unfortunately, a great deal to do with architecture, but scarcely enough to keep an architect fully employed, or to warrant the inclusion of an architect among its numbers; and the same may be said of an engineer. What is meant by a layman it is difficult to understand; presumably he is not to be either a doctor, lawyer, an architect, or an engineer, and we do not know whether men of other occupations are to be ruled out. It is difficult to say what useful function can be performed on the Board of Control by a gentleman who has not had any training whatever in any business or profession. And it is still more difficult to discover what his department is to be.

With the second recommendation we are in hearty agreement. The medical superintendents of asylums are for the most part no longer medical. Their time is chiefly occupied in administrative duties, in looking after the ventilation, and the drains, and the farm, and the piggeries; in making returns to the Board of Control, in filling up forms, in making entries in books, and in signing numerous documents, and making innumerable regulations for the staff and the patients under their control. As long as the chief officer of an asylum, who is supposed to be a medical officer, and in charge of the treatment of the patients, is thus occupied, the treatment of mental diseases is not likely to advance. As a matter of fact it is stagnant, and has remained stagnant for many years. The superintendent is himself precluded from making researches into the treatment of his patients, and he is not inclined to welcome researches on the part of his subordinates, which would tend to eclipse his own merits and his own position. The position is a wrong one, and the sooner it is changed the better.

Another matter upon the agenda is that the payments, salaries, and emoluments, of officers in asylums should be standardised as far as possible. This is all very well, but it is very much more important that another step should be taken, which does not appear upon the agenda. The most important reform needed in the administration of asylums is reform in the selection of the medical officers. When the superintendency of an asylum falls vacant a number of applications are received from various parts of the country, and from gentlemen who are quite unknown to the Visiting Committee, and who must be appointed without personal knowledge, and upon merely written testimonials. The appointment does not necessarily, it may be said that it does not usually, fall to the candidate who is best qualified. It falls to the gentleman who can produce the best testimonials, that is to say, whose friends and official superiors are most experienced in writing testimonials, and know what qualifications are likely to be most valued by a Visiting Committee. Amongst those qualifications eminence in the medical profession, professional learning, and professional skill do not occupy a high place. Some Visiting Committees value economical administration above all other qualifications. Most of them attach, as in present circumstances they are bound to do, chief importance to administrative ability; but professional attainments are very little considered.

The conveners of the conference are not blind to this state of stagnation, for they say in their invitation that they are unanimous in the view that the present Lunacy Laws were passed with little or no regard to securing the proper treatment in cases of incipient insanity. They might have gone further and said that the Lunacy Laws were passed with little or no regard to securing a proper treatment in any cases of insanity. The chief purpose of the present Lunacy Laws is to safeguard the liberty of the subject, to secure that no person shall be certified or detained as a madman unless he actually is mad. To these all other considerations are subservient, and the notion that madness can be cured or that anything is needful in the treatment of mad people, except safeguarding them from cruelty, evidently never entered into the mind of legislators.

The conference is invited to express in the strongest possible terms its opinion that the Lunacy Laws are effete, and that a drastic amendment of them is of urgent national importance. We quite agree that this is so, and at a time of general reconstruction, and when old ideas on all subjects are being revised, there is perhaps some chance that the view of three generation ago, that all that is necessary in Lunacy Legislation is to safeguard the liberty of the subject, may at last be superseded.

The conference is evidently swayed considerably by the opinions of its medical members, and therefore, we find that one of the matters placed in the very forefront of the agenda is a change of names. Nothing will ever enlighten the alienists of this country on this point. Their fixed determination is that if they can only induce people to call madness by some other name, it will be madness no longer, and that if they can change the name of asylum to the ridiculous title of mental hospital, they will have achieved a great feat, and will have effected an important reform. Already they have done much in this direction. For years past they have called adolescent insanity by the name of *dementia præcox*, and having done so they sit down in contentment, and admire the portentous achievement, which assumes in their eyes the rank of a most important discovery. The conference would abolish the words lunatic and lunacy; it does not indicate what titles shall be substituted for these time-honoured terms. They have at least the merit that they are not misleading, for, although they no longer bear their original signification either in law or in medicine, they are usually taken to mean neither more nor less than madmen and madness.

The term "person of unsound mind" although in legal fiction it is held to mean the same as madness does not in fact mean the same, and "mental disorder" is a very different thing indeed from madness. If the conference is so weak as to be swayed by its medical members who have these absurd notions about words, and object to the terms lunatic and lunacy, it has only two alternatives before it. It may either go back to the old terms "madmen and madness," or it must borrow from Volapuk or Esperanto some entirely new term. If it flounders among "mental diseases" and "mental hospitals," it will, in the first place, be erroneous, and in the second place hold itself up to ridicule from those who are accustomed to look into the meaning of words.

